# Vibration-Assisted Femtosecond Laser Drilling with Controllable Taper Angles for AMOLED Fine Metal Mask Fabrication

**DOI:** 10.3390/ma10020212

**Published:** 2017-02-21

**Authors:** Wonsuk Choi, Hoon Young Kim, Jin Woo Jeon, Won Seok Chang, Sung-Hak Cho

**Affiliations:** 1 Department of Nano-Mechatronics, UST, Korea University of Science and Technology, 217 Gajeong-Ro, Yuseong-Gu, Daejeon 34113, Korea; cws@kimm.re.kr (W.C.); hykim@kimm.re.kr (H.Y.K.); 2 Department of Laser & Electron Beam Application, KIMM, Korea Institute of Machinery and Material, 156 Gajeongbuk-Ro, Yuseong-Gu, Daejeon 34103, Korea; jwjeon@kimm.re.kr; 3 Department of Nanomechanics, KIMM, Korea Institute of Machinery and Material, 156 Gajeongbuk-Ro, Yuseong-Gu, Daejeon 34103, Korea

**Keywords:** taper angle control, femtosecond laser, hole drilling, Invar plate, AMOLED

## Abstract

This study investigates the effect of focal plane variation using vibration in a femtosecond laser hole drilling process on Invar alloy fabrication quality for the production of fine metal masks (FMMs). FMMs are used in the red, green, blue (RGB) evaporation process in Active Matrix Organic Light-Emitting Diode (AMOLED) manufacturing. The taper angle of the hole is adjusted by attaching the objective lens to a micro-vibrator and continuously changing the focal plane position. Eight laser pulses were used to examine how the hole characteristics vary with the first focal plane’s position, where the first pulse is focused at an initial position and the focal planes of subsequent pulses move downward. The results showed that the hole taper angle can be controlled by varying the amplitude of the continuously operating vibrator during femtosecond laser hole machining. The taper angles were changed between 31.8° and 43.9° by adjusting the vibrator amplitude at a frequency of 100 Hz. Femtosecond laser hole drilling with controllable taper angles is expected to be used in the precision micro-machining of various smart devices.

## 1. Introduction

Active Matrix Organic Light-Emitting Diode (AMOLED) technology has opened a new world of display possibilities because of its advantages of thinness, bendability, foldability, transparency, and a wide color gamut [1,2,3]. Because of these advantages, AMOLEDs are being considered for high-resolution displays suitable for virtual reality displays and various other applications. The production of fine metal masks (FMMs), which is essential in red, green, blue (RGB) evaporation processes [4,5,6], is important for the development of high-resolution displays because the pixel size in the RGB evaporation process is directly associated with the FMM pattern size. Thus, in order to produce higher-resolution displays, FMM patterns must be smaller. FMMs are usually made of Invar alloy. Because Invar alloys have an extremely low thermal expansion coefficient [7,8], they are used in precision tools and watch parts, as well as in the RGB evaporation process with FMMs that need dimensional stability during temperature changes. At present, FMMs with a pattern size of 30 μm are produced by the chemical etching process [9], but a smaller pattern size is needed for high-resolution displays [10,11]. However, using this technique it is difficult to form a pattern smaller than the material thickness, because chemical etching occurs in an isotropic manner. Furthermore, the taper angle of the pattern cannot be controlled. It is difficult to produce thin FMMs because of the warping, melting, and alignment problems that occur during the evaporation process. In addition, the chemical etching process is destructive to the environment, has a high equipment cost, is complicated, and is potentially harmful to human health [12,13,14].

To overcome the disadvantages of chemical etching, femtosecond laser processing is attracting increased attention. Femtosecond lasers have an advantage over nanosecond lasers in ultra-precision processing in terms of the small heat-affected zone (HAZ) owing to their short pulse duration [15,16]. Productivity and process quality control for femtosecond laser FMM machining concerns a deep pulse processing depth for the same process variables and adjustment of the taper angle of the machined hole.

According to previous research [17,18,19,20,21,22], laser processing can control the taper angle of a pattern, but it has been difficult to control the taper angle with pattern sizes of 10 to 20 μm using a single independent parameter. Park et al. [23] processed holes with high aspect ratios in bulk copper by means of a femtosecond laser with 25,000 pulses using a vibrator. However, no research has been reported regarding the effects on productivity of varying the processing depth for a small number of pulses by changing the focal position of the objective lens using a vibrator. Additionally, the interaction between a femtosecond laser and Invar alloy has not yet been investigated.

This study proposes a femtosecond laser hole drilling system using a vibrator. To achieve high productivity, the change in machining depth between the first pulse focus plane and subsequent pulse focus planes is investigated using a small number of pulses. Furthermore, the change in hole taper angle caused by a change in vibrator amplitude is studied in order to control processing quality.

## 2. Experimental Setup

**Figure 1:**
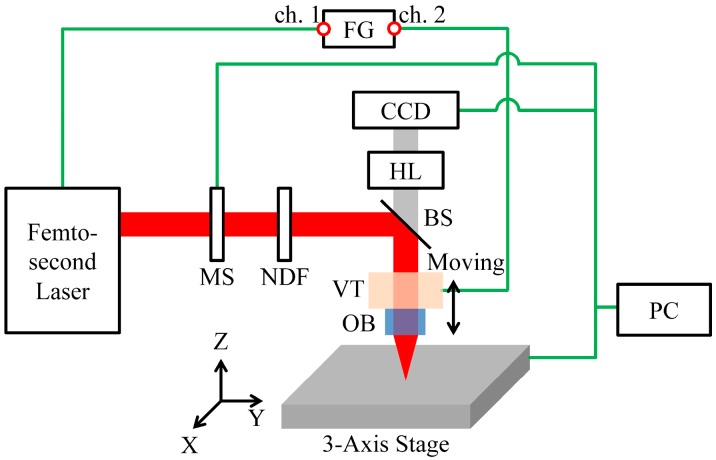
Schematic of the vibration-assisted femtosecond laser machining system. (MS: Mechanical shutter, NDF: Neutral density filter, BS: Beam splitter, VT: Vibrator, OB: Objective lens, CCD: Camera, HL: Halogen lamp, FG: Function generator).

Figure 1 shows a schematic diagram of the femtosecond laser hole drilling system using a vibrator. A regenerative amplifier-type femtosecond laser based on Yb:KGW with a central wavelength of 1027 nm, average power of 6 W, pulse duration of 190 fs, repetition rate of single shot ~200 kHz, maximum pulse energy of 1 mJ, and M^2^ value of 1.3 (Model No. Pharos SP, Light conversion, Lithuania) was used in this study. The vibrator-based femtosecond laser hole drilling system consisted of a femtosecond laser, beam delivery optics, attenuator, a three-axis stage, a co-axial vision system, a co-axial illuminator, an objective lens, a vibrator, a function generator, an amplifier, and an oscilloscope. An M Plan Apo NIR lens (Mitutoyo, Kawasaki, Japan) was used for the focusing optic. The lens has a magnification of 50×, effective focal length of 4 mm, 0.42 NA, laser beam diameter at the lens of approximately 3.5 mm, and DOF of 1.6 μm. The objective lens was mounted on a vibrator (Physik Instrumente, Karlsruhe, Germany) to change the focal position quickly and accurately during processing. The signal from the function generator (NF Corporation, Yokohama, Japan) was transferred to the vibrator through the amplifier. The oscilloscope (Tektronix, Beaverton, OR, USA) monitored the input values and the actual motion to check and correct the frequency, amplitude, and delay.

Experimental samples were prepared with Invar, which is an alloy composed of Fe (64%) and Ni (36%), at a sample thickness of 13 μm. Because the samples were thin, defocusing may be caused by deflection and other unfavorable effects may arise in processing. Thus, the samples were pulled in four directions to make them as flat as possible. Furthermore, the samples were floated over a fixed bulk substrate to ensure femtosecond laser pulse penetration without damaging the back side of the samples. The front and back surfaces of the samples were cleaned before and after the experiments to prevent errors caused by contaminants.

## 3. Experimental Results and Discussion

**Figure 2:**
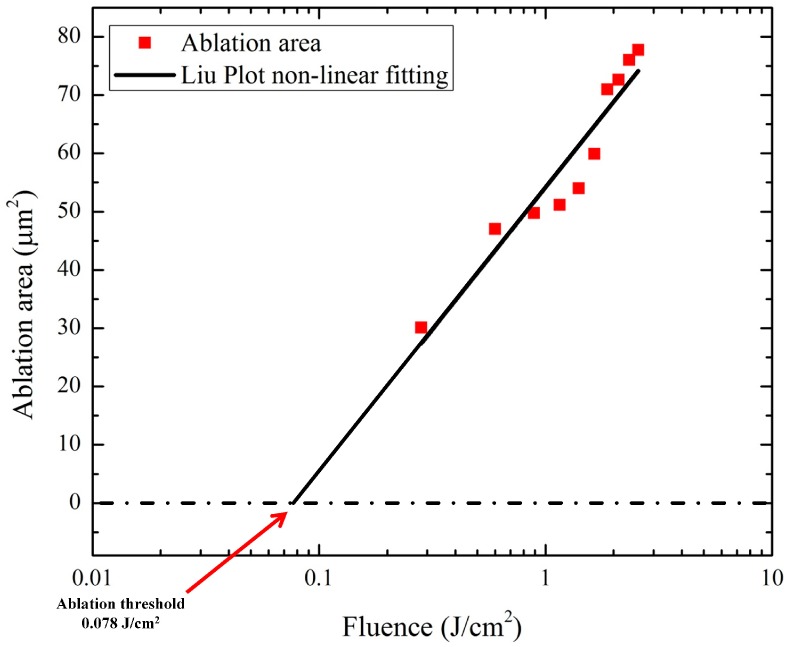
Invar ablation threshold experimental results and calculation (Liu plot non-linear fitting).

Material removal in laser machining occurs when the laser fluence is higher than the material's ablation threshold, which is strongly dependent on the interaction between light and matter. To test this using a small number of pulses, it is necessary to use a Liu plot [24,25] to determine the threshold at which ablation takes place. The fluence needed to penetrate a sample is higher than this threshold. The minimum fluence at which machining takes place is calculated by extrapolating the relationship between the fluence and the hole diameter on a Liu plot. To do this, the objective lens is accurately focused on the sample surface and each pulse is generated while increasing and decreasing the fluence. The processed sample is then observed with an optical microscope. The diameter of each processed part is calculated and associated with the fluence at that position. The nonlinear relationship between the fluence and hole diameter is shown in Figure 2. The ablation threshold value was calculated from the interaction between the Invar alloy and the femtosecond laser.

The calculated ablation threshold (0.078 J/cm^2^) was used to determine the minimum fluence limit. Additionally, the fluence was increased from the ablation threshold until the sample was penetrated with 8 pulses. The fluence was measured as 112.59 J/cm^2^. In this study, the objective lens was moved along the optical axis by a micro-vibrator and the position of the focus plane was changed continuously. When the focus plane changed, the spot size and the ablation depth also changed. Thus, the processing diameter and depth can be varied using the initial position of the pulse focal plane and the change of focal position for subsequent pulses. As the number of pulses increases, the variation in processing caused by this change decreases. However, because one oscillation cycle and a small number of pulses are used, an experiment must be first conducted to determine the effects of these factors on processing. Therefore, the focal plane of the pulses and its vibrator-induced movement are determined using the pulse energy measured from the Liu plot while taking into account the oscillation frequency and laser pulse repetition rate. The experimental parameters were established considering the initial position of the pulse focal plane and its change in position. The focal plane was fixed at each position by manually controlling the vibrator, and the laser beam was irradiated for a set number of pulses. The machining depth was determined by observing either the penetration of the processing hole or the diameter of the exit hole. Experimental parameters for the optimum machining depth were determined and applied to control the taper angle by adjusting the amplitude of vibration. The taper angle control experiment applied a sinusoidal function to the vibrator to continuously vibrate it, irradiating the sample with a set number of laser pulses. The taper angle was then calculated by measuring the entrance and exit diameters of the processed hole.

**Figure 3:**
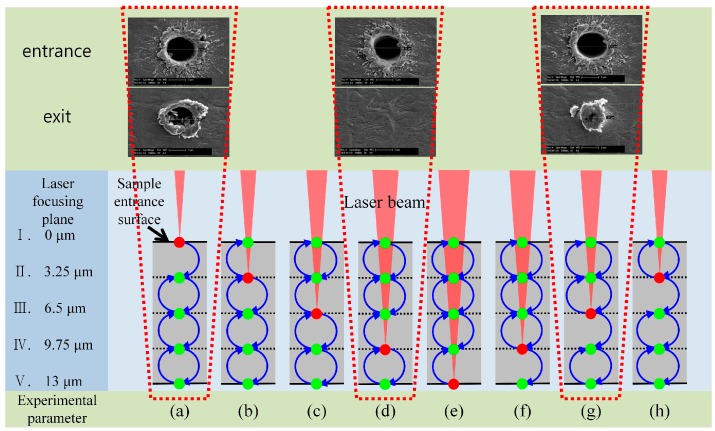
Schematic diagram of the experimental procedure and typical scanning electron microscope images of the entrance and exit holes. Red dots indicate the first pulse while green dots indicate the remaining 2–8 pulses. The blue arrows indicate the focus direction of motion (e.g., parameter a: I → II → III → IV → V → IV → III → II).

Figure 3 shows a schematic diagram of the experiment for determining the process parameters of the vibrator along with the scanning electron microscopy (SEM) image of the front and back sides of the processed hole. The vibration amplitude was set to 13 μm, which was the same size as the sample thickness, in order to adjust the taper angle according to the amplitude (as described later). Eight pulses accumulated at one hole per vibration cycle at this oscillation frequency and laser repetition rate. Considering that the eight pulses were focused and converted into sinusoidal functions by vibration, five focal planes including the sample front and back sides were defined by dividing the material into four parts along its thickness (Figure 3, I–V). Furthermore, the parameters of the experiment were determined by considering the number of focal points (Figure 3, I–V) at which the first pulse was focused when the focus was changed by the vibration and the number of subsequent focal plane positions (Figure 3a–h). The red dot in each experimental parameter indicates the position at which the first pulse was focused, and from which position it moved after the vibration started. The focal plane moved in the direction of the blue arrow according to the experimental parameters, and the second to eighth pulses were focused on the green points in each of the specified focal planes. The entrance diameters of the processed holes seem to be similar for each experimental parameter. However, the exit diameters were significantly affected by the experimental parameters, and some samples did not have an exit diameter because they were not penetrated. The results show that experiment (a) was the optimum processing condition with the deepest processed depth.

Using a smaller number of pulses is advantageous because productivity is improved as a result of the shorter processing time. Furthermore, the heat accumulation caused by the overlapping of many low-energy pulses is greatly reduced. It is believed that the processed depth of each of the eight pulses must be as large as possible in order to obtain deep processing depths from machining. Because the processed depth of each pulse varies and the spot size of the focused pulses varies according to the depths of the previous pulses and the displacement of the focus plane caused by the vibrator, the accumulated depth processed by eight pulses is affected by the experimental parameters. The magnitude of this phenomenon may be large or small depending on the depth of focus (DOF) of the lens used, which also seems to be strongly influenced by the location of the pulse-focusing planes.

**Figure 4:**
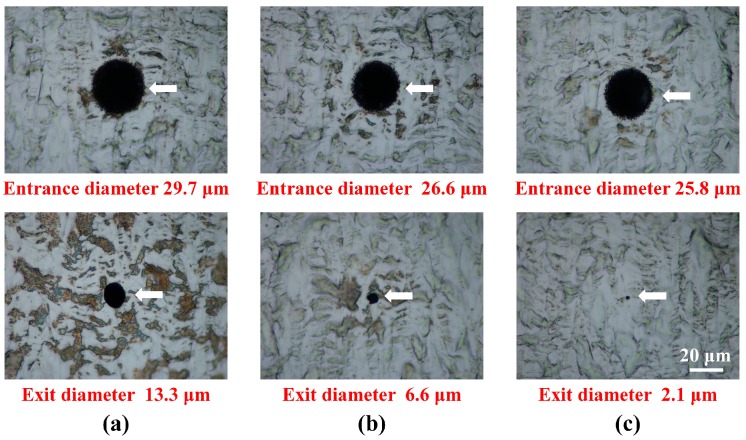
Typical images of a demonstration of vibration-assisted femtosecond laser hole drilling with taper angle control using vibration amplitudes of (**a**) 13 μm; (**b**) 6.5 μm; and (**c**) 1.3 μm.

**Figure 5:**
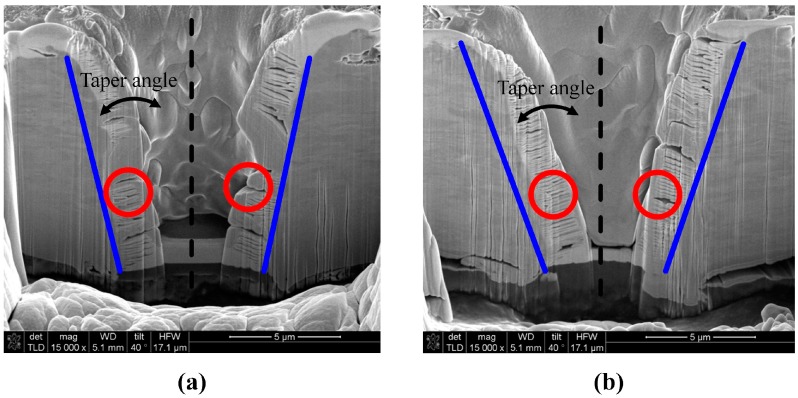
Examples of cross-sectional images of the hole drilled by vibration-assisted femtosecond laser drilling: Taper angles (**a**): 10.03°; (**b**): 14.22°; Experimental parameters: 1027 nm wavelength, 190 fs pulse duration, 1 kHz repetition rate, 1.1 μJ pulse energy, 100 pulses. Settings for M Plan Apo NIR (Mitutoyo): 0.42 NA, 50× lens magnification, 200 Hz of vibration frequency, vibration amplitudes (**a**): 1.4 μm; (**b**): 3.1 μm.

**Figure 6:**
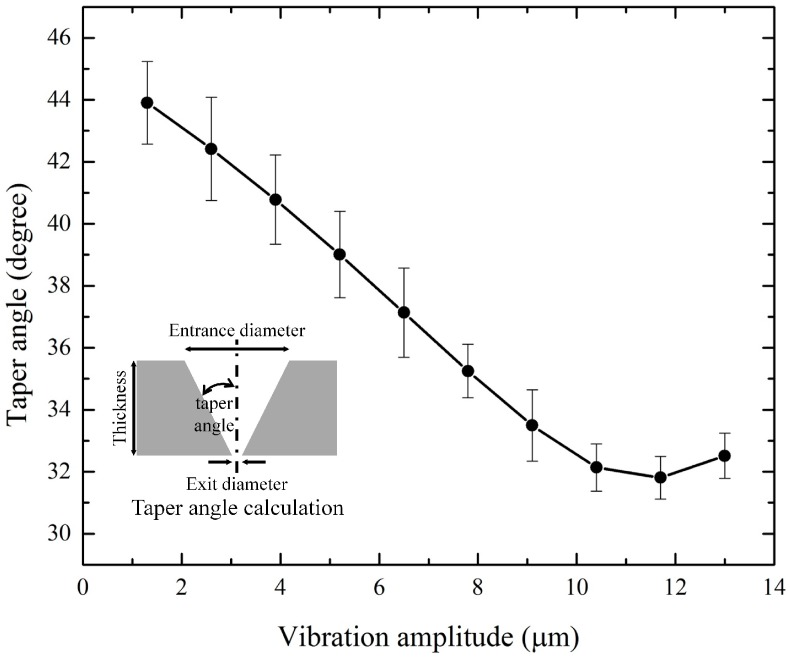
Plot of hole taper angle via vibration amplitude control using a vibration-assisted femtosecond laser hole drilling system. Inset: the definition of the taper angle.

Focusing the first pulse on the sample surface (Figure 3a) was found to provide the best efficiency under the same processing conditions. These results were used in the vibration amplitude variation for controlling the hole taper angle. A laser fluence of 235.34 J/cm^2^ was used, which was approximately twice that of the previous experiment because the sample might not be penetrated if the vibration amplitude was changed. We adjusted the phase difference in the 2-channel timing synchronization by applying the results of the previous experimental parameter (a). This is because the ‘time delay’ between the voltage application and initial operation was expected to be different for each vibrator and laser. By adjusting the phase difference between the two channels, we set the vibrator to move as soon as the first pulse was focused on the sample surface. Additional experiments were performed to determine the phase difference. Experiments were performed using a laser repetition rate of 900 Hz, laser fluence of 235.34 J/cm^2^, vibration frequency of 100 Hz, and burst mode of 8 pulses. Figure 4 shows optical images of the entrance and exit in the experiment where the taper angle was varied based on vibration amplitude. It can be seen that both the entrance and exit diameters changed as the amplitude changed. However, the exit diameter changed rapidly compared to the entrance diameter, which caused the taper angle of the processed hole to change. Figure 5 shows examples of cross-sectional images of the hole drilled by vibration-assisted femtosecond laser drilling. Blue lines are the interfaces of the drilled holes and red circles are platinum coated areas, which were made for the focused ion beam measurement. Figure 6 shows the change in taper angle caused by adjustment of the vibration amplitude along with the definition of the taper angle. The taper angle was calculated as a trigonometric function using the entrance diameter, exit diameter, and sample thickness. Although the result contains some errors, it can be concluded that the smaller the amplitude, the smaller the diameter of the exit, and the larger the taper angle. In addition, when the amplitude was reduced by around 13 μm, the taper angle decreased slightly before increasing again. 

The differences in results processed under the same conditions are expected because the thin material can easily bend and the pulse energy of each pulse can differ slightly. In order to reduce these errors, the samples need to be attached or vacuum-adsorbed to the substrate, and the pulse energy must be calibrated through feedback control so as to induce uniform energy in every pulse if possible.

## 4. Conclusions

The effects of the pulse focal plane locations on machining efficiency and hole taper angle were investigated using a femtosecond laser machining system with a continuously oscillating micro-vibrator. An experiment was conducted using eight beam pulses of fluence 112.59 J/cm^2^ each. It was found that the processing efficiency and productivity were high when the first pulse was focused on the top surface of the sample because the processed depth was the deepest. This indicates that synchronizing the laser and vibrator in the hole processing system can affect productivity. In this study, hole machining with an adjustable taper angle in the range of 31.8°–43.9° was successfully demonstrated by controlling the amplitude of the continuously oscillating vibrator. It was found that, as the amplitude of the vibrator decreased, the taper angle of the processed hole tended to increase. Femtosecond laser hole drilling using a vibrator capable of controlling the taper angle is expected to be useful for the nano/micro processing of solar cells and the manufacturing of FMMs, which are used in the AMOLED manufacturing process.
